# Focus on patients in medical education

**DOI:** 10.3205/zma001493

**Published:** 2021-06-15

**Authors:** Marjo Wijnen-Meijer

**Affiliations:** 1 Technical University of Munich, School of Medicine, TUM Medical Education Center, Munich, Germany

## Editorial

Making medical treatment decisions can be difficult for both patients and physicians. Patients are increasingly expected and asked to be involved in health care decisions. Both patients and physicians should possess communication skills and certain qualities that make patient participation possible. Patients should be more insistent and involved and physicians should be less authoritarian in order to shift to shared decision-making and patient-centred medical care [1].

In the systematic review of Brom et al. [1] the congruence between patients’ preferences and their perceived participation in medical decision-making is explored. Several studies have investigated factors associated with preferred role in medical decision-making. Younger patients, higher educated people and women more frequently prefer a more active role in decision-making while older people were found to more often prefer a ‘paternalistic attitude’ from their physician [1], [2]. The stage of illness also may influence patient preferences regarding participation. For example, a study among patients with prostate cancer showed that they preferred a more active role later in their disease process, assumingly because of getting used to being ill. Moreover, patients with cancer more often desired a passive role than non-cancer patients and in general, patients more often desired a passive role when asked about more specific treatment option [1].

Studies showed that active participation was associated with positive health outcomes such as overall quality of life, higher physical and social functioning and less fatigue. Nonetheless some studies showed that pushing patients into active participation in medical decisions could have negative consequences such as decisional regret, increased anxiety, doubt whether they have made the right treatment decision and unnecessary stress [1], [3]. The review of Brom et al. [1] suggests that a similar approach to all patients is not likely to meet patients’ wishes, since preferences for participation vary among patients. 

Cegala et al. [4] showed that physicians provided more information overall and more information in response to patients’ questions when communicating with active patients than when interacting with passive patients. The high patient participation during a medical interview helps the physician to more accurately understand the patient’s goals, interests, and concerns, thus allowing the physician to address matters that are important to the patient, provide explanations and offer reassurance [4].

It is important to include patients actively in medical education to train in advance the future doctors how to interact with patients, develop communication skills and offer patient-centered care. In order to develop clinical skills medical students should be exposed to real clinical cases, settings and patients but at the same time it should not interfere with patients’ rights. Carmody et al. [5] evaluated patients’ perceptions of medical students involved in their care at a women’s hospital. Most patients were satisfied with student involvement in care and believed students should be part of the clinical routine. 

The study of Chang et al. [6] explored the implementation of longitudinal integrated clerkships (LICs) in Taiwan which contributed to patient-centeredness and identified values such as care facilitation, companionship, and empathy. LIC students offered care facilitation by serving as a bridge between the physicians and patients, by reminding, consulting, tracking disease progression, and researching solutions for problems. Wittkampf et al. [7] explored medical students’ learning experiences, within a longitudinal program where students followed patients in an out-of-hospital setting and suggested that following patients in their home environment for a prolonged period supports the development of meaningful relationships between students and patients and prompts patient-centredness.

Further studies showed that generally patients perceive positively medical students and acknowledged the educational benefit of involving them in the medical care [8], [9]. 

Gordon et al. [10] reviewed different researches on involving patients in the teaching of medical students and pointed out several benefits. Firstly for learners: their understanding of patientcentered care, applying their physical examination, consultation and history-taking skills, being aware of the impact of illness on everyday life, the effect on partners and families and patient empowerment. The benefits for patients include satisfaction from using their personal experiences in medical education and greater confidence in their knowledge of their own health or illness. Patients agree to be teaching subjects because they want to help and perceive it as an expression of altruism [11]. 

Henriksen and Ringsted [12] indicated that patient-instructors sessions create learning environment where content matter is accompanied by realism and individual perspectives on diseases. The teaching format is characterised by authenticity and intimacy and be conductive to asking questions and making mistakes. Isaacson et al. [13] studied patient perceptions of having 1^st^- and 2^nd^-year medical students involved in their ambulant care. Most patients would want to see a student again and a high percent of patients felt the presence of a student added value to their visit as it increased the time and the quality of the visit.

Patient involvement in medical education may improve training of the medical workforce and the associated healthcare benefit for the population. As students gain more experience with patients, including being involved in shared decision making and learning to acknowledge and respect their feelings and choice, it is expected that patients will ultimately benefit [14].

In several articles described in this issue, patients are directly or indirectly involved in medical training. 

The article von Demmer et al. [15] describes how practical examinations with ambulant patients can be implemented. The article by Glässel et al. [16] describes the importance of using real patient experiences in the development of OSCEs. At the time of the COVID-19 Pandemic, contact with patients by medical and dental students is limited. Although this problem cannot be solved completely, Crome et al. [17] describe how diagnostic skills can be trained, by the use of digital patient cases in education. An important prerequisite for shared decision making in clinical practice is the ability to assess the knowledge of the patients. The study by Harendza et al. [18] on the knowledge of laypersons about the anatomical locations or organs and the definitions of commonly used medical terms, is relevant in this context.

A focus on patients and their experiences in medical education is crucial for understanding and strengthening patient-physician relationship and providing a competent individual approach to every different case. 

## Competing interests

The author declares that she has no competing interests.
